# Patterns of Non-Administration of Ordered Doses of Venous Thromboembolism Prophylaxis: Implications for Novel Intervention Strategies

**DOI:** 10.1371/journal.pone.0066311

**Published:** 2013-06-14

**Authors:** Kenneth M. Shermock, Brandyn D. Lau, Elliott R. Haut, Deborah B. Hobson, Valerie S. Ganetsky, Peggy S. Kraus, Leigh E. Efird, Christoph U. Lehmann, Brian L. Pinto, Patricia A. Ross, Michael B. Streiff

**Affiliations:** 1 Department of Pharmacy, The Johns Hopkins Hospital, Baltimore, Maryland, United States of America; 2 Department of Epidemiology, The Johns Hopkins Bloomberg School of Public Health, Baltimore, Maryland, United States of America; 3 Division of Acute Care Surgery, Department of Surgery, The Johns Hopkins University School of Medicine, Baltimore, Maryland, United States of America; 4 Department of Anesthesiology and Critical Care Medicine, The Johns Hopkins University School of Medicine, Baltimore, Maryland, United States of America; 5 Department of Emergency Medicine, The Johns Hopkins University School of Medicine, Baltimore, Maryland, United States of America; 6 Division of General Internal Medicine, Department of Medicine, The Johns Hopkins University School of Medicine, Baltimore, Maryland, United States of America; 7 Division of Hematology, Department of Medicine, The Johns Hopkins University School of Medicine, Baltimore, Maryland, United States of America; 8 The Armstrong Institute for Patient Safety, Johns Hopkins Medicine, Baltimore, Maryland, United States of America; 9 Department of Pathology, The Johns Hopkins University School of Medicine, Baltimore, Maryland, United States of America; 10 Division of Health Sciences Informatics, Department of Pediatrics, The Johns Hopkins University School of Medicine, Baltimore, Maryland, United States of America; University of New South Wales, Australia

## Abstract

**Background:**

Recent studies have documented high rates of non-administration of ordered venous thromboembolism (VTE) prophylaxis doses. Intervention strategies that target all patients have been effective, but prohibitively resource-intensive. We aimed to identify efficient intervention strategies based on patterns of non-administration of ordered VTE prophylaxis.

**Methods and Findings:**

In this retrospective review of electronic medication administration records, we included adult hospitalized patients who were ordered pharmacologic VTE prophylaxis with unfractionated heparin or enoxaparin over a seven-month period. The primary measure was the proportion of ordered doses of VTE prophylaxis not administered, assessed at the patient, floor, and floor type levels. Differences in non-administration rates between groups were assessed using generalized estimating equations. A total of 103,160 ordered VTE prophylaxis doses during 10,516 patient visits on twenty-nine patient floors were analyzed. Overall, 11.9% of ordered doses were not administered. Approximately 19% of patients missed at least one quarter and 8% of patients missed over one half of ordered doses. There was marked heterogeneity in non-administration rate at the floor level (range: 5–27%). Patients on medicine floors missed a significantly larger proportion (18%) of ordered doses compared to patients on other floor types (8%, Odds Ratio: 2.4, p<0.0001). However, more than half of patients received at least 86% of their ordered doses, even on the lowest performing floor. The 20% of patients who missed at least two ordered doses accounted for 80% of all missed doses.

**Conclusions:**

A substantial proportion of ordered doses of VTE prophylaxis were not administered. The heterogeneity in non-administration rate between patients, floors, and floor types can be used to target interventions. The small proportion of patients that missed multiple ordered doses accounted for a large majority of non-administered doses. This recognition of the Pareto principle provides opportunity to efficiently target a relatively small group of patients for intervention.

## Introduction

Venous thromboembolism (VTE), comprised of deep venous thrombosis (DVT) and/or pulmonary embolism (PE) represent a serious public health challenge, affecting up to 600,000 Americans annually. [1], [2] The consequences can be deadly; VTE has been identified as the most common cause of preventable mortality in hospitalized patients, accounting for up to 10% of hospital deaths. [3], 4 Despite widespread understanding of the prevalence of VTE and the efficacy of prophylactic therapy, numerous studies have documented that VTE prophylaxis is underprescribed. [5]–[11] Accordingly, the Agency for Healthcare Research and Quality and the Centers for Medicare and Medicaid Services (CMS) have prioritized improvement in VTE prophylaxis practice [2], [12]–[14].

Recent technology-based initiatives have increased the proportion of patients who are assessed for VTE risk and who are prescribed VTE prophylaxis. [15], [16] However, we recognize that ordering appropriate therapy does not ensure its administration. Studies of mechanical VTE prophylaxis have documented high non-adherence rates ordered intermittent pneumatic compression devices. [17], [18] Fanikos, et al., in a study of 250 hospitalized patients, found that approximately 10% of ordered pharmacologic VTE prophylaxis doses were not administered. [19] Patient refusal was the most commonly documented reason for non-administration, accounting for 44% of missed doses of VTE prophylaxis. In response, Piazza et al. devised an intervention wherein a research pharmacist conducted an education program for all patients prescribed VTE prophylaxis therapy on medical and surgical units. [20] While this intervention was seemingly effective, it was very labor-intensive, requiring one hour per patient and was applied indiscriminately, even to those receiving all ordered doses.

We anticipated that a larger data set could provide useful information regarding variation in the pattern of non-administered doses. Understanding the factors associated with non-administered doses may lead to a more effective and efficient intervention. The objective of the current study was to identify the extent of and patterns in the non-administration of ordered pharmacologic VTE prophylaxis in order to inform decision making regarding efficient intervention strategies.

## Methods and Materials

Approval was obtained from the Institutional Review Board of the Johns Hopkins Medical Institutions and an informed consent waiver was granted. The Johns Hopkins Hospital recently completed implementation of an integrated computer system to facilitate electronic prescribing, dispensing, and administration of medications. During the admission process and upon a transition from one level of care to another, our computerized provider order entry (CPOE) system prompts the prescriber to answer questions regarding each patient’s risk for a VTE event. Based on the patient-specific answers to these questions, a clinical decision support tool (CDS) automatically recommends a pharmacologic VTE prophylaxis regimen, which may be easily selected or modified by the prescriber. [15] After a regimen is selected, a pharmacist electronically verifies it, and the order populates to the electronic medication administration record (eMAR) to notify nursing staff to administer the regimen. For each scheduled medication dose, a nurse records whether the dose was given or not and, if not, the reason why the dose was omitted.

We conducted a retrospective review of electronic medication administration record (eMAR) and computerized provider order entry system (CPOE) data. We included records from all hospitalized patients aged 18 years or older who received prescriber orders for one of the following pharmacologic VTE prophylaxis regimens from December 1, 2007 through June 30, 2008: unfractionated heparin (UFH) 5000 units or 7500 units given subcutaneously every 8 or 12 hours or subcutaneous enoxaparin 40 mg every 24 hours or 30 mg every 12 hours. Data obtained directly from the CPOE database included patient demographics, orders details and order tasks details (dose administration records). The study occurred during the implementation phase of our CPOE system; only patients on floors with the CPOE system functioning were included in this study.

### Statistical Analysis

The proportion of ordered doses not administered was calculated at the patient, floor, floor type, and regimen levels as: (the total number of ordered doses not administered)/(the total number of ordered doses). Floor and floor type were defined as the discharge location. Patterns of non-administered doses were assessed to improve our understanding of the phenomenon and to identify effective, rational intervention strategies. Differences in the non-administration rate between groups were assessed by generalized estimating equations (GEE), to account for correlation of response variables within individuals. For these models, a binomial distribution, logit link, and autoregressive correlation structure were chosen. For assessment of binary outcomes that were not longitudinal, the chi-squared test for comparison of proportions analysis was used. Relative risk (RR) and 95% confidence intervals (CIs) were also calculated for these analyses. A p-value of <0.05 was considered significant for all analyses. All analyses were conducted using STATA v.11 (College Station, TX) statistical analysis software.

## Results

A total of 103,160 pharmacologic VTE prophylaxis doses were ordered during 10,516 patient visits. Patients from 29 floors were included in this analysis, including 11 medicine floors, 9 surgery floors, 4 neurology floors, and 5 intensive care units. Baseline characteristics for the patients included in our study can be found in Table 1. The frequency of different pharmacologic VTE prophylaxis dosing regimens is shown in Table 2. The most common VTE prophylaxis regimen was UFH 5000 units every 8 hours, comprising 57% of all prophylaxis orders. Overall, 16% of all orders were for an enoxaparin regimen. Enoxaparin use was most common on surgery floors, which accounted for over 57% of all enoxaparin use.

**Table 1:** Demographics of patient visits from December 1, 2007 through June 30, 2008.

Medical Service Type(number of patient visits)	Medicine(n = 4829)	Surgery(n = 3849)	Neurology(n = 1599)	ICU(n = 239)
**Unique Patients**	3797	3308	1460	236
**Mean age (SD), years**	54 (17)	54 (17)	53 (17)	57 (16)
**Female n (%)**	2,493 (51.6%)	1,899 (49.3%)	851 (53.2%)	89 (37.2%)
**Race n (%)**				
Black	1,601 (33.2%)	941 (24.4%)	416 (26.0%)	95 (39.7%)
Caucasian	3,075 (63.7%)	2,599 (67.5%)	1,087 (68.0%)	129 (54.0%)
Other	153 (3.2%)	309 (8.0%)	96 (6.0%)	15 (6.3%)
**Insurance status n (%)**				
Private				
Blue Cross	441 (9.1%)	837 (21.7%)	384 (24.0%)	43 (18.0%)
Commercial	257 (5.3%)	460 (12.0%)	174 (10.9%)	13 (5.4%)
Government				
Medicare	1,850 (38.3%)	1,214 (31.5%)	473 (29.6%)	95 (39.7%)
Medicaid	1,710 (35.4%)	522 (13.6%)	187 (11.7%)	54 (22.6%)
Managed Care Payer	490 (10.1%)	667 (17.3%)	345 (21.6%)	28 (11.7%)
Self Pay	37 (0.8%)	31 (0.8%)	12 (0.8%)	2 (0.8%)
Other	44 (0.9%)	118 (3.1%)	24 (1.5%)	4 (1.7%)

**Table 2:** Total number of VTE prophylaxis doses ordered, not administered, and documented as not administered due to patient refusal.

	Total Number ofDoses Ordered	Number of Ordered Doses NotAdministered (% of total)	Number of Doses Not Administered and Documented as Refused (% of Not Administered)
**All Doses**	103,160	12,239 (11.9%)	7,217 (59.0%)
Medication			
UFH	86,958	11,161 (12.8%)*	6,630 (59.4%)†
Enoxaparin	16,202	1,078 (6.7%)	587 (54.5%)
**Dose and Frequency**			
UFH 5000 units every 8 hours	58,299	6,852 (11.8%)*	3,813 (55.6%)*
UFH 5000 units every 12 hours	28,159	4,278 (15.2%)	2,798 (65.4%)
Enoxaparin 40 mg every 24 hours	12,211	876 (7.2%)	502 (57.3%)
Enoxaparin 30 mg every 12 hours	3,991	202 (5.1%)	85 (42.1%)
UFH 7500 units every 8 hours	500	31 (6.2%)	19 (61.3%)
**Race**			
Black	43,081	5,793 (13.5%)*	3,586 (61.9%)*
Caucasian	54,123	5,670 (10.5%)	3,147 (55.5%)
Hispanic/Asian/Other	5,956	776 (13.0%)	484 (62.4%)
**Sex**			
Female	51,305	6,085 (11.9%)	3,684 (60.5%)*
Male	51,855	6,154 (11.9%)	3,533 (57.4%)
**Hospital Floor Type**			
Medicine	41,000	7,177 (17.5%)*	4,833 (67.3%)*
Surgery	42,299	3,567 (8.4%)	1,778 (49.8%)
Neurology	16,488	1,245 (7.4%)	564 (45.3%)
ICU	3,373	250 (7.6%)	42 (16.8%)

* p<0.0001.

† p = 0.002.

The total number of doses, number of ordered doses not administered and the number of doses documented as refused are given in Table 2, by various drug regimen and demographic/clinical characteristics. Of the 103,160 ordered VTE prophylaxis doses, 12,239 (12%) were not administered (Table 2). The proportion of ordered doses not administered was significantly higher on medicine floors (17.5%) compared with surgery, neurology, and ICU floors (8.1%; OR: 2.1; 95% CI: 2.0–2.2). Likewise, the proportion of doses not administered and documented as refused was significantly higher on medicine floors compared with the other floor types (11.8% vs. 3.8%, OR: 3.1, 95% CI: 2.9–3.2). Overall, ordered doses of UFH were significantly more likely to not be administered (13% versus 7%; OR: 1.9; 95% CI: 1.8–2.0) and documented as refused (8% vs. 4%, OR: 2.1; 95% CI: 1.9–2.3) compared with enoxaparin. This phenomenon does not appear to be related to the frequency of administration, as heparin regimens ordered every 12 hours were three times more likely to not be administered compared with enoxaparin regimens ordered at the same frequency (15% vs. 5%, OR: 3.0, 95% CI: 2.6–3.4). This was true on virtually every unit, regardless of unit type, that had substantial use of both every 12 hour regimens. Floor type was strongly associated with non-administration rate independent of regimen type, with medicine floors having a significantly higher non-administration rate for the three most common regimens:

- UFH 5,000 units every 8 hours (17% vs. 9%, OR: 1.9, 95% CI: 1.8–2.2),
- UFH 5,000 units every 12 hours (24% vs. 9%, OR: 2.7, 95% CI: 2.5–2.8), and
- enoxaparin 40 mg every 24 hours (9% vs. 6%, OR: 1.6, 95% CI: 1.4–1.8).

There was no statistical difference in non-administration rate between floor types for the other regimens. The proportion of ordered doses not administered was significantly higher overall for African American patients (14%) compared with Caucasians (11%, OR: 1.28; 95% CI: 1.24, 1.33). However, this relationship was confounded by the distribution of patients between the floor types. African Americans represented nearly two-thirds (63%) of patients on medicine floors (i.e., the floors with significantly higher non-administration rates). On medicine floors, African Americans had a significantly *lower* rate of non-administration (17%) compared with Caucasians (19%, OR: 0.9; 95% CI: 0.86–0.95).

Patient or family member refusal was the most commonly documented reason for non-administration of ordered doses, accounting for 59% of all non-administrations. It was significantly more common for ordered doses to be documented as refused on medicine floors (12% of all ordered doses) compared with surgery (4%, p<0.001) or other types of floors (3%, p<0.001). Documented refusals accounted for two-thirds (67%) of non-administered doses on medicine floors, and approximately half on surgery (50%) and neurology (45%) floors (p<0.001).

**Figure 1:**
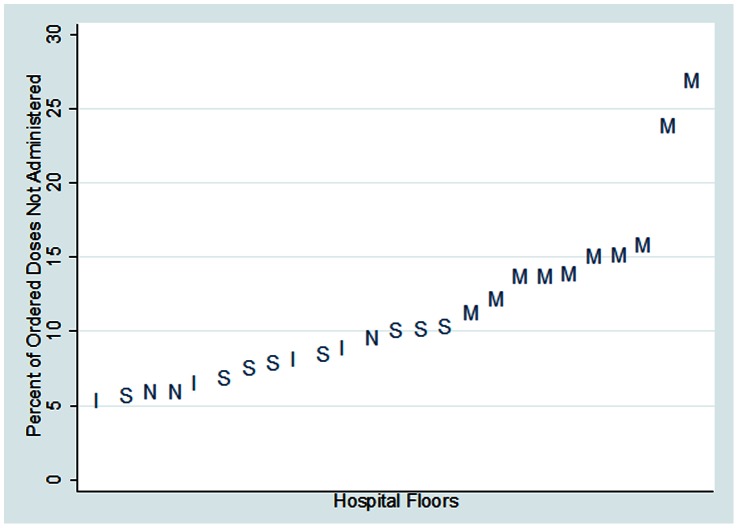
Proportion of ordered VTE prophylaxis doses not administered by floor, with floor type indicated, (M = medicine, S = surgery, N = neurology, I = ICU).

When non-administration was examined at the floor level, there was nearly five-fold variation in the proportion of ordered doses not administered, ranging from a high of 26.9% to a low of 5.4% (see Figure 1). Floor type was strongly associated with the non-administration rate. Of the twenty-five floors, medicine floors accounted for the ten highest non-administration rates. No medicine floors were associated with the fifteen lowest rates. Figure 1 reveals two medicine units appear as outliers, with substantially higher non-administration rates of 27% and 24%. Non-administration rates among the medicine floors varied by more than two-fold, from 26.9% to 11.3%. There was a smaller degree of variation between surgery floors (10.4% to 5.7%), neurology floors (9.6% to 5.9%), and intensive care units (8.9% to 5.4%).

Overall, only 59% percent of patients received all ordered doses of VTE prophylaxis. Approximately 19% of patients missed at least one quarter and 8% of patients missed over one half of ordered doses. An additional 5% missed over three quarters of their ordered doses. Patients on medicine floors were significantly more likely to miss at least one dose (46% of patients), compared to patients from all other study units (36%, OR: 1.53; 95% CI: 1.42–1.66). Likewise, patients on medicine floors were significantly less likely to receive at least 90% of their ordered doses (62% of patients) compared with patients from all other units (76% of patients; OR: 0.52; 95% CI: 0.47–0.56). At the other end of the spectrum, patients on medicine floors were significantly more likely to miss more than 50% of their ordered doses (13% vs. 4%, OR: 3.52; 95% CI: 3.01–4.12). Finally, patients on medicine floors were significantly more likely to miss at least 80% of ordered doses (8.2% vs. 2.5%; OR: 3.49; 95% CI: 2.86–4.28).

**Figure 2:**
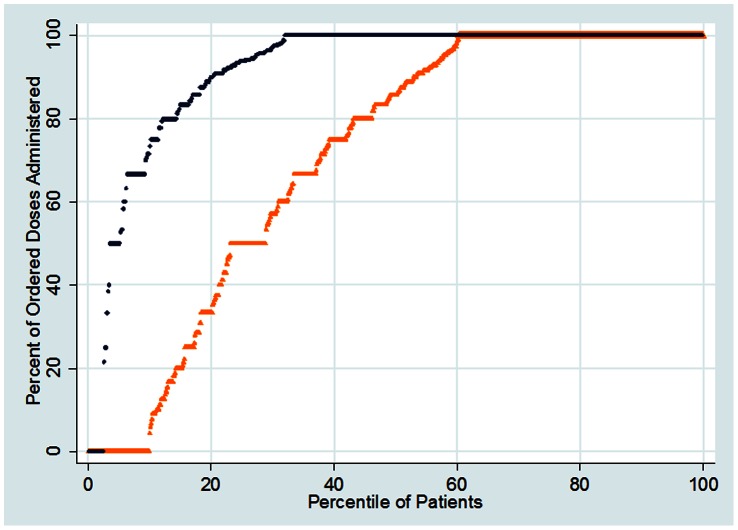
Percentile plot of the percent of ordered doses that were administered for each patient on the patient floor with the highest overall administration rate (black) and the patient floor with the lowest overall administration rate (orange).

Figure 2 is a percentile plot of the percent of ordered doses that were administered to each patient on the floors with the highest and lowest overall administration rate. It allows identification of the percent of patients at or below a given percent of ordered doses administered. For example, approximately 20% of patients received ninety percent or less of ordered doses on one floor (top series of points). Conversely, 80% of patients on this floor received at least ninety percent of ordered doses. In comparison, 50% of patients on the other floor (bottom series of points) received ninety percent or less of ordered doses. Likewise, 50% of patients, a much lower percent than the top-performing floor, received ninety percent or more of ordered doses. This plot also highlights the difference between the floors in the percent of patients that received none of their ordered doses: less than 5% of patients on the top-performing floor compared with approximately 10% of patients on the other. Despite these differences, it is notable that a substantial percent (more than half) of patients received at least 86% of their ordered doses, even on the lowest performing floor.

The realization that most patients receive at least 80% of ordered doses, even on low-performing floors, led us to search for an efficient marker to identify patients for potential intervention. The Pareto principle generally applied when using the number of ordered doses not administered for each patient as a signal. The approximately 20% of patients who missed at least two ordered doses accounted for 80% of the total number of ordered doses not administered (Table 3). This relationship held across floors and floor types (Table 3).

**Table 3:** Number of patients and the number of all ordered doses not administered, by total of ordered doses not given during the patient visit.

Overall				
Total Number of Ordered Doses Not Administered During Patient Visit	Number (%) of All Patients, N = 10,516		Number (%) of All Ordered Doses Not Administered, N = 12,239	
At least 1	4,265 (40.6%)		12,239 (100%)	
At least 2	2,071 (19.7%)		10,045 (82.1%)	
At least 3	1,214 (11.5%)		8,331 (68.1%)	
At least 4	819 (7.8%)		7,146 (58.4%)	
*Medicine*	n = 4,829			
At least 1	2,229 (46.2%)		7,177 (100%)	
At least 2	1,214 (25.1%)		6,162 (85.9%)	
At least 3	752 (15.6%)		5,238 (73.0%)	
At least 4	524 (10.9%)		4,554 (63.5%)	
*Surgery*		n = 3,849		
At least 1		1,379 (35.8%)		3,567 (100%)
At least 2		573 (14.9%)		2,761 (77.4%)
At least 3		299 (7.8%)		2,213 (62.0%)
At least 4		197 (5.1%)		1,907 (53.5%)
*Neurology*		n = 1,599		
At least 1		545 (34.1%)		1245 (100%)
At least 2		235 (14.7%)		935 (75.1%)
At least 3		129 (8.1%)		723 (58.1%)
At least 4		81 (5.1%)		579 (46.5%)
*ICU*		n = 250		
At least 1		112 (46.9%)		250 (100%)
At least 2		49 (20.5%)		187 (74.8%)
At least 3		34 (14.2%)		157 (62.8%)
At least 4		17 (7.1%)		106 (42.4%)

## Discussion

Our analysis of a relatively large data set led to the following insight: 1) a substantial proportion (11.9%) of ordered doses of VTE prophylaxis were not administered, 2) certain individual floors and floor types (i.e., medicine floors) had significantly higher non-administration rates than others, and 3) the Pareto principle applied in that the small proportion of patients that missed multiple ordered doses (∼20%) accounted for a large majority (∼80%) of non-administered doses. These insights have fundamentally changed our understanding of the issue of non-administered VTE prophylaxis doses and will lead us towards research approaches and intervention strategies that we did not originally anticipate. The Pareto principle provides an opportunity to efficiently target a relatively small group of patients for intervention. Perhaps prioritizing specific floors and floor types with the highest non-administration rates for further study and/or intervention is a better use of scarce quality improvement resources.

This study found nearly 12% of ordered doses of pharmacologic VTE prophylaxis were not administered, nearly identical to rates reported in a recent report by another research team.^19^ The similarity in rates between these institutions leads us to believe the problem may not be unique to our hospital, but is likely widespread. We agree with other investigators that this overall rate is unacceptably high. However, our dataset was large and robust enough to expose a surprising level of heterogeneity in the non-administration rates between floors, floor types, and patients. This realization led us to understand this issue to be more intricate and nuanced than previously thought. While the overall rate is concerning, the non-administration of ordered doses of VTE prophylaxis is highly concentrated on medicine floors. Our ten medicine floors accounted for the ten highest non-administration rates. Furthermore, certain floors within floor type were identified as clear outliers. This was most dramatically displayed by the two medicine floors that had non-administration rates of approximately 25%. These realizations have led us to the next round of questions: what is different about the units with very high rates? Are the important drivers of non-administered doses patient-related, provider-related, a combination, or none of the above? What is the deeper story behind the high rate of patient refusals? Does a culture of care develop on a floor that facilitates non-administration of ordered doses? To what extent could the non-administration of ordered doses be considered rational? What strategies can be developed to close the gap, no matter what the key determinants are found to be?

The fact that relatively small numbers of patients account for a large majority of non-administered doses has obvious implications for intervention strategies. Previous investigators have provided evidence of success in decreasing non-administration rates by providing education to all patients – but at great cost that is likely unsustainable. [20] We have identified a signal that should be available in nearly real-time – non-administration of multiple ordered doses for the same patient – that could greatly increase the efficiency of any chosen intervention. Across all floors, the Pareto principle applied; the small proportion of patients (∼20%) who had multiple non-administered ordered doses accounted for a significant majority (∼80%) of all missed doses. Understanding this pattern will enable us to identify and prioritize the approximately 20% of patients who missed at least one quarter of their ordered doses and approximately 10% of the patients who missed over one half of their ordered VTE prophylaxis doses. Perhaps equally important, most patients received at least 86% of their ordered doses, even on floors with the lowest overall non-administration rate. These facts, taken together, suggest that visiting with every patient is unnecessary and a more efficient, targeted approach is indicated. One potential way to address the problem would be the design of dashboards that show in real time the rate of non-administered doses per unit. A separate, but important point is that an intervention strategy centered on visiting with patients assumes the primary drivers of non-administration lie with them. This remains unproven and warrants further scrutiny.

Heparin regimens had higher rates of non-administration and documented patient refusal. This phenomenon was independent of hospital floor type and frequency of administration. For example, while medicine floors had significantly higher overall rates of non-administration and documented patient refusals, heparin regimens had significantly higher non-administration and documented refusal rates than enoxaparin regimens on medicine floors. Likewise, on virtually every floor that had substantial use of both heparin and enoxaparin regimens ordered every 12 hours, these rates were significantly higher for the heparin regimens. We can currently offer no explanation for these observations, but we have launched initiatives to further investigate.

This study critically evaluates administration rates of medication for VTE prevention only, but highlights the need to further explore administration patterns for other types of medication. A study of medication dose omissions in a UK hospital found that over the course of 7 days, 12.4% of all medication doses were not administered and that “patient refused drug” was the documented rationale in 45.4% of cases.

Several limitations of our study deserve consideration. Due to the retrospective nature of the study, we were unable to ascertain the context and deeper reasons for non-administration of ordered doses. For example, we did not have access to information regarding the provider responsible for administration of the doses or his/her role. Therefore, we do not understand to what extent non-administered doses tends to cluster within provider. Additionally, due to limitations of the date-time stamp in our data set, the sequential nature of ordered doses could not be accounted for analytically. Future data sets should be assembled so that the longitudinal nature of non-administered doses can be assessed. The stated reasons for non-administration came largely via a dropdown list in the eMAR. Undoubtedly, much important information is lost in the translation. The best intervention will correspond to the true nature of the problem, a nature we do not yet fully understand. Our short term goal is to resist any temptation to rush to judgment while beginning a multi-disciplinary dialogue to consider the evidence and potential intervention strategies. Another important limitation is that each patient’s location in the data sets we used corresponded to the discharge location. This could introduce some inaccuracy regarding non-administration rates by individual floor. This limitation is probably most significant for the intensive care floors. Another limitation of our study was that we were only able to evaluate patients on floors with CPOE, although this accounted for nearly 95% of all admissions.

In conclusion, we ascertained patterns in the non-administration of ordered VTE prophylaxis doses that are important, unexpected, and actionable. While we implement intervention strategies based on current evidence, we intend to launch an aggressive research campaign to fill existing knowledge deficits. From a quantitative perspective, we intend to explore other potentially important sources of variation at the patient, nurse, and floor levels. We will investigate the relative importance of floor-based effects (suggesting the importance of a “culture of care”) versus patient-specific effects. From a qualitative perspective, we need to develop deeper understanding from the perspectives of all relevant stakeholders, including patients, nurses and prescribers. Our ongoing goal is to continually refine intervention strategies so they align with the true nature of the challenge and produce more effective and efficient solutions.
